# A Scalable Bioreactor Platform for Reproducible Production and Characterization of Ovarian Cancer-Derived Extracellular Vesicles

**DOI:** 10.3390/bioengineering13080896

**Published:** 2026-08-05

**Authors:** Wei Fu, Kalpana Deepa Priya Dorayappan, Colin Hisey, Lakshmi Narasimhan Chakrapani, Sydney Wiggins, Shyam Sundaram, Zachary Lambert, Kim Truc Nguyen, Sudhiksha Anbu Chelian, Eduardo Reategui, Karuppaiyah Selvendiran, Derek J. Hansford

**Affiliations:** 1 Department of Biomedical Engineering, The Ohio State University, Columbus, OH 43210, USA; fu.1066@osu.edu (W.F.); colin.hisey@northwestern.edu (C.H.); sydneywiggins2029@u.northwestern.edu (S.W.); zlambert6746@gmail.com (Z.L.); 2 Department of Obstetrics and Gynecology, Division of Gynecologic Oncology, The Ohio State University Wexner Medical Center, Columbus, OH 43210, USA; kalpanadeepapriya.dorayappan@osumc.edu (K.D.P.D.); lakshminarasimhan.chakrapani@osumc.edu (L.N.C.); sundaram.81@buckeyemail.osu.edu (S.S.); 3 William G. Lowrie Department of Chemical and Biomolecular Engineering, The Ohio State University, Columbus, OH 43210, USA; vickyings@gmail.com (K.T.N.); reategui.8@osu.edu (E.R.); 4 Department of Biomedical Engineering, Northwestern University, Evanston, IL 60208, USA; 5 Department of Health Sciences, The Ohio State University, Columbus, OH 43210, USA; anbuchelian.1@buckeyemail.osu.edu

**Keywords:** extracellular vesicles, bioreactor culture, serum-free culture, ovarian cancer-related cell lines, CELLine AD 1000, nanoparticle tracking analysis, imaging flow cytometry, tetraspanins, size-exclusion chromatography

## Abstract

Extracellular vesicles (EVs) from ovarian cancer cells are valuable sources for candidate biomarker studies, but conventional static flask culture yields limited material and is difficult to scale reproducibly. We evaluated a serum-free CELLine AD 1000 bioreactor workflow for producing EVs from four ovarian cancer-related (OC-related) cell lines (OVCAR4, CaOV3, PA1, SW626) and human dermal fibroblasts (HDFa) as a non-cancer control. Cells were adapted to CDM-HD serum-free medium and maintained for eight weeks with twice-weekly conditioned-medium collection. EVs were isolated by differential ultracentrifugation followed by size-exclusion chromatography and characterized by nanoparticle tracking analysis, imaging flow cytometry, Western blotting, and transmission and scanning electron microscopy. Across longitudinal harvests, OC-related cultures generally produced higher EV particle concentrations and A280-based bulk protein estimates than HDFa, while individual cell lines showed distinct production profiles and membrane-associated growth patterns. A parallel OVCAR4 T-175 flask, maintained in its original serum-containing medium, provided a contextual reference indicating higher per-collection EV particle recovery with the bioreactor, although this was not a matched culture-format comparison. EV-enriched preparations contained vesicle-like particles, with modal diameters of approximately 96–128 nm. Using imaging flow cytometry, the CD9 signal was higher in OC-related EVs and CD63 was most prominent in HDFa; CD9 and CD63 were also detected in OC-related EV lysates by Western blotting. Because one bioreactor was operated per cell line, these findings should be interpreted as preliminary and descriptive rather than statistically comparative. Overall, this study provides a practical serum-free CELLine AD 1000 workflow for generating characterized OC-related EV material for downstream analytical studies.

## 1. Introduction

Ovarian cancer (OC) remains the most lethal gynecological malignancy, with an estimated 324,000 new cases and over 200,000 deaths worldwide in 2022 [1], and an estimated 20,890 new cases and 12,730 deaths projected for the United States in 2025 [2]. Because early symptoms are non-specific and no effective population-level screening test is currently recommended for average-risk women, most patients are diagnosed at advanced stages, for which long-term survival remains poor [3]. Established serum markers such as CA-125 lack the sensitivity and specificity required for early detection [4], and large randomized screening trials based on CA-125 and transvaginal ultrasound have not reduced OC mortality [5,6]. These limitations have intensified interest in liquid biopsy, and extracellular vesicles (EVs) have drawn attention as a potential source of tumor-derived biomarkers [7].

Extracellular vesicles (EVs) are nanoscale, membrane-enclosed particles released by virtually all cell types and can carry proteins, lipids, and nucleic acids reflecting the state of their parent cells [8,9,10,11]. They mediate intercellular communication and participate in tumor processes including angiogenesis, immune modulation, and metastasis [12]. In OC, tumor-derived EVs carry cancer-associated surface markers and microRNA signatures with diagnostic potential [13,14,15,16,17] and can promote disease progression and chemoresistance [18,19]. However, developing EV-based assays and mechanistic studies requires sufficient quantities of well-characterized EV material, which is difficult to obtain from patient biofluids alone.

Patient-derived biofluids provide clinically relevant material but are heterogeneous and limited in volume, which constrains reproducibility and downstream analysis [20,21,22,23,24]. Well-characterized OC cell lines offer a controlled complementary source of tumor EVs for assay development, workflow optimization, and controlled comparison across experimental conditions, in line with current ISEV recommendations for rigorous EV characterization (MISEV2023) [25,26]. The choice of culture platform and medium, however, can affect EV yield, purity, and downstream interpretation.

Conventional adherent culture in tissue-culture flasks is poorly suited to large-scale EV production: it requires large surface areas and media volumes yet yields EV quantities that are often insufficient for proteomic, transcriptomic, or functional studies [27,28]. Reliance on serum-containing media also introduces contaminating bovine EVs that confound characterization [29,30], and variability among serum lots can further reduce consistency [30]. These constraints motivate serum-free culture approaches that can generate sufficient EV material under defined conditions.

Bioreactor systems can overcome part of this limitation by maintaining cells at high density over extended periods, enabling continuous EV harvest with higher yield and lower labor than static flasks [27,31,32,33,34,35,36,37]. The CELLine AD 1000 is a compact, dual-compartment bioreactor that separates the cell chamber from the nutrient reservoir with a 10 kDa semipermeable membrane, supporting high-density culture and concentrated EV collection in a format suitable for standard cell culture conditions, while limiting bovine EV contamination of the conditioned medium [27,31,38]. Studies in cancer cell-line and mesenchymal stromal cell models have reported approximately 8- to over 100-fold increases in EV yield relative to flask culture, along with improvements in workflow efficiency [28,34,35,39]. Despite these advances, bioreactor-based EV production has not been reported for ovarian cancer-related cell lines.

In this study, we evaluated a serum-free bioreactor workflow based on the CELLine AD 1000 for EV production from four ovarian cancer-related cell lines (OVCAR4, CaOV3, PA1, and SW626), with human dermal fibroblasts (HDFa) as a non-cancer control. SW626 was originally isolated from the ovary but is now considered likely to represent an ovarian metastasis of a primary colon adenocarcinoma [40]; we therefore include it as an ovary-derived metastatic comparator rather than as a representative ovarian cancer cell line. Cells were adapted to serum-free, chemically defined medium and maintained for eight weeks, during which EV yield, A280-based bulk protein estimates, tetraspanin markers (CD9 and CD63), vesicle morphology, and long-term culture stability were assessed. We aimed to determine whether this workflow could generate sufficient, well-characterized OC-related EV material for downstream analytical studies.

## 2. Materials and Methods

### 2.1. Cell Lines and Maintenance Culture

The ovarian cancer-related cell lines OVCAR4, CaOV3, PA1, and SW626, and human dermal fibroblasts (HDFa), were obtained from the American Type Culture Collection (ATCC, Manassas, VA, USA). HDFa served as a non-cancer control. Under the culture conditions used in this study, cells were maintained in T-175 flasks (Corning, Corning, NY, USA) prior to serum-free transition: OVCAR4 in RPMI-1640 (Gibco, Thermo Fisher Scientific, Waltham, MA, USA) and CaOV3, PA1, and SW626 in DMEM (Gibco), each supplemented with 10% fetal bovine serum (FBS; Gibco; heat-inactivated for PA1) and 1% penicillin–streptomycin (Gibco). HDFa were maintained in DMEM supplemented with 10% FBS and 1% penicillin–streptomycin. All cultures were incubated at 37 °C in a humidified atmosphere containing 5% CO_2_.

### 2.2. Serum-Free Adaptation and Bioreactor Culture

Cells harvested from ten T-175 flasks of each cell line at 80–90% confluence were pooled to inoculate one CELLine AD 1000 bioreactor (WHEATON, Millville, NJ, USA). Before bioreactor inoculation, each cell line was adapted to serum-free DMEM/F12 supplemented with CDM-HD serum replacement (FiberCell Systems, Frederick, MD, USA) using a line-specific stepwise medium-transition schedule. Briefly, cultures were gradually transitioned from their original FBS-containing complete medium to DMEM/CDM-HD and then to DMEM/F12/CDM-HD through intermediate formulations, typically including DMEM/FBS, 50% DMEM/FBS plus 50% DMEM/CDM-HD, DMEM/CDM-HD, 50% DMEM/CDM-HD plus 50% DMEM/F12/CDM-HD, and DMEM/F12/CDM-HD. The formulation was advanced once per week, allowing progressive replacement of FBS with CDM-HD and transition of the basal medium to DMEM/F12.

For PA1, CaOV3, SW626, and HDFa, the adaptation process generally required five weeks. OVCAR4, which was originally maintained in RPMI-1640 supplemented with FBS, included an additional RPMI-to-DMEM transition step and required approximately seven weeks in total. HDFa were processed using the same serum-free adaptation and bioreactor workflow as a non-cancer control.

After adaptation, bioreactors were maintained in DMEM/F12 supplemented with 10% CDM-HD for up to eight weeks as the production phase. Conditioned medium from the cell compartment was collected twice weekly, with approximately 15 mL harvested per bioreactor at each collection. The nutrient reservoir was replenished with fresh medium at each harvest.

To provide a reference for conventional T-175 flask culture, an OVCAR4 flask was maintained in parallel in its original pre-adaptation medium, RPMI-1640 supplemented with 10% FBS. Conditioned medium was collected once weekly, with 15 mL collected per harvest. This comparison was included as a contextual reference rather than as a controlled assessment of medium composition, because the flask and bioreactor conditions differed in culture format, medium formulation, cell density, and harvest schedule.

### 2.3. Extracellular Vesicle Isolation

Conditioned medium was processed by sequential differential centrifugation at 4 °C: 250× *g* for 5 min and 2000× *g* for 10 min to remove cells and debris; 20,000× *g* for 30 min to deplete large EVs; and 100,000× *g* for 60 min to obtain a small EV (sEV)-enriched pellet. Ultracentrifugation steps were performed on a Beckman Coulter Optima XPN ultracentrifuge with a 50.2 Ti fixed-angle rotor (k-factor 69). The sEV-enriched pellet was resuspended in 500 µL phosphate-buffered saline (PBS) and applied to a qEVoriginal 35 nm size-exclusion chromatography (SEC) column (Izon Science Ltd., Christchurch, New Zealand) to further separate EVs from soluble protein contaminants [41]. EV-enriched fractions were collected according to the recommended collection window [31], pooled (∼1.6 mL total), and stored at −80 °C in Eppendorf Protein LoBind^®^ tubes (Eppendorf SE, Hamburg, Germany). All subsequent characterization was performed on these SEC fractions.

### 2.4. Nanoparticle Tracking Analysis

Particle concentration and size distribution were measured using a NanoSight NS300 system (Malvern Panalytical, Malvern, UK) [42]. EV-enriched SEC fractions were diluted in 0.22-µm-filtered PBS prior to analysis, using line-specific dilution factors based on sample preparation records. CaOV3, SW626, and HDFa samples were analyzed at 100-fold dilution, whereas OVCAR4 and PA1 samples were analyzed at 1000-fold dilution. Five 30-s videos were captured per sample under continuous syringe-pump flow. Acquisition settings, including camera level and detection threshold, were adjusted as needed according to sample signal intensity; detection thresholds ranged from 5 to 10 across samples. Data were analyzed using NanoSight NTA software v3.3. Particle concentration, mean diameter, modal diameter, D10, D50, and D90 values were recorded for each sample. For longitudinal concentration comparisons, NTA-reported particle concentrations were manually corrected using the corresponding dilution factor. Size metrics were reported directly from the NTA output and were not dilution-corrected.

### 2.5. A280-Based Protein Estimation

Bulk protein content of SEC-purified EV-enriched preparations was estimated using a NanoDrop One spectrophotometer (Thermo Fisher Scientific) by absorbance at 280 nm, with PBS as the blank. For each measurement, 1–2 µL of sample was loaded onto the NanoDrop pedestal. A280 values were recorded as bulk protein estimates and were not interpreted as EV-specific protein concentrations. Protein values were recorded in mg/mL and converted to µg/mL for graphical presentation where indicated.

### 2.6. Imaging Flow Cytometry

EV surface markers were analyzed on an Amnis^®^ ImageStream^®^X Mk II imaging flow cytometer (Luminex Corporation, Austin, TX, USA). EVs were labeled with EXO-GLOW Emerald Green as a general EV dye, detected in the FITC channel, together with anti-CD9-PE and anti-CD63-AF647 (BioLegend, San Diego, CA, USA), and incubated for 1 h at 4 °C in the dark. EXO-GLOW events were gated as EV-enriched events, and CD9-positive and CD63-positive events were quantified within this gate. Unstained vesicle controls were used to define background fluorescence and gating boundaries. Data were analyzed using IDEAS software version 6.2, and event concentrations were reported as objects/mL.

### 2.7. Western Blotting

EV and cell lysates were prepared in RIPA buffer (Thermo Fisher Scientific) with protease and phosphatase inhibitors (Roche, Basel, Switzerland). Samples normalized by A280-estimated protein content were separated on 10% SDS-PAGE gels and transferred to PVDF membranes (MilliporeSigma, Burlington, MA, USA). Membranes were blocked in 5% non-fat milk in TBS-T and probed overnight at 4 °C with anti-CD9 (1:1000), anti-CD63 (1:1000), and anti-GAPDH (1:2000; Cell Signaling Technology, Danvers, MA, USA), followed by HRP-conjugated secondary antibodies (1:5000) and ECL detection on a ChemiDoc system (Bio-Rad, Hercules, CA, USA). Band intensities were quantified using ImageJ2, version 2.16.0/1.54p (National Institutes of Health, Bethesda, MD, USA).

### 2.8. Transmission Electron Microscopy

EV morphology was assessed by negative-stain transmission electron microscopy (TEM). Two 20-µL droplets of water for injection (WFI) and two 20-µL droplets of negative stain (UranyLess EM Stain; Electron Microscopy Sciences, Hatfield, PA, USA) were placed on a strip of Parafilm M (Amcor, Neenah, WI, USA). Carbon Type B, 200-mesh thick nickel TEM grids (Ted Pella, Inc., Redding, CA, USA) were plasma-treated for 1 min using a PE-50 plasma cleaner (Plasma Etch, Inc., Carson City, NV, USA). Subsequently, 10 µL of the purified EV suspension was carefully applied to the treated surface of each grid and incubated for 1 min. Excess liquid was gently removed using filter paper.

The EV-coated grids were sequentially immersed in the two WFI droplets and blotted with filter paper after each wash. The grids were then immersed in the first negative-stain droplet, blotted, and transferred to the second stain droplet. After incubation for approximately 22 s, excess stain was gently wicked away using filter paper. The stained grids were placed in a grid box and air-dried overnight at room temperature. TEM imaging was subsequently performed using a Tecnai TF-20 transmission electron microscope (FEI Company, Hillsboro, OR, USA) operated at an accelerating voltage of 200 kV.

### 2.9. Scanning Electron Microscopy

After eight weeks of culture, bioreactor membranes were processed for SEM to examine membrane surface morphology and cell attachment. Membranes were fixed in 4% glutaraldehyde, post-fixed in 1% osmium tetroxide, dehydrated through a graded ethanol series, and critical-point dried (Tousimis Autosamdri-931; Tousimis Research Corp., Rockville, MD, USA). Samples were mounted on aluminum stubs, sputter-coated with gold-palladium, and imaged. Representative images were acquired at multiple magnifications at 5.00 kV in ETD secondary-electron mode to assess membrane coverage, cell attachment, and growth morphology.

### 2.10. Data Analysis and Descriptive Statistics

Data analysis and visualization were performed using Python v3.14.0 (Python Software Foundation, Wilmington, DE, USA) and Matplotlib, version 3.10.7. Quantitative data are presented as mean ± standard deviation (SD), unless otherwise indicated. Because one CELLine AD 1000 bioreactor was operated for each cell line, repeated harvests across the production period were treated as longitudinal within-run measurements rather than independent biological replicates. Comparisons among cell lines and culture formats were therefore descriptive, and no formal inferential statistical testing was performed unless explicitly stated in the corresponding figure legend. Error bars represent variability across harvests or measurements, as specified in each figure legend.

## 3. Results

### 3.1. Longitudinal EV Particle and Bulk Protein Output During Bioreactor Culture

**Figure 1:**
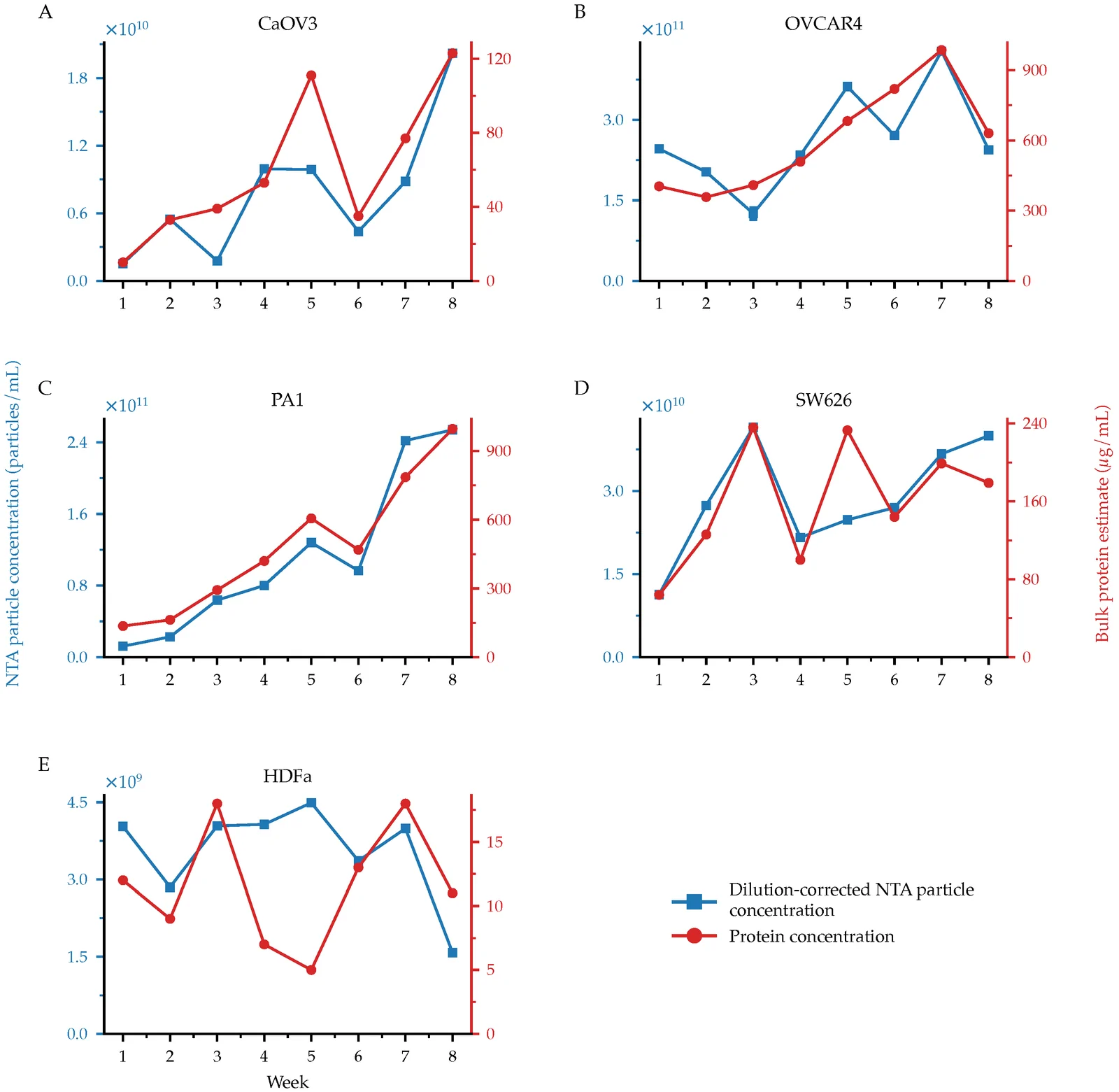
EV particle concentration and A280-based bulk protein output during bioreactor culture. Panels represent (**A**) CaOV3, (**B**) OVCAR4, (**C**) PA1, (**D**) SW626, and (**E**) HDFa. EV particle concentration (blue) and A280-based bulk protein estimate (red) are shown for one representative harvest per week over an eight-week culture period for four OC-related cell lines (OVCAR4, CaOV3, PA1, SW626) and one non-cancer control (HDFa). OVCAR4 and PA1 showed increasing output over time, whereas CaOV3 and SW626 showed greater week-to-week variation; HDFa showed the lowest values. Data represent repeated harvests from one bioreactor per cell line and are shown as longitudinal within-run measurements.

Four OC-related cell lines (OVCAR4, CaOV3, PA1, SW626) and the HDFa control were cultured in independent CELLine AD 1000 bioreactors following stepwise adaptation to serum-free CDM-HD medium and monitored over eight weeks. Cultures were maintained for repeated twice-weekly conditioned-medium collection over the production period. HDFa produced lower particle and A280-based bulk protein values than the OC-related cell lines across the collection period (Figure 1). OVCAR4 and PA1 showed increasing particle and A280-based bulk protein output over the production period; CaOV3 reached relatively high values but showed week-to-week variation; SW626 showed moderate output with variability between consecutive harvests; HDFa showed the lowest particle and bulk protein values. Particle concentration and A280-based bulk protein estimates showed similar temporal trends in most cultures.

### 3.2. Comparison of OVCAR4 Particle Recovery Between Bioreactor and Conventional Flask Culture

**Figure 2:**
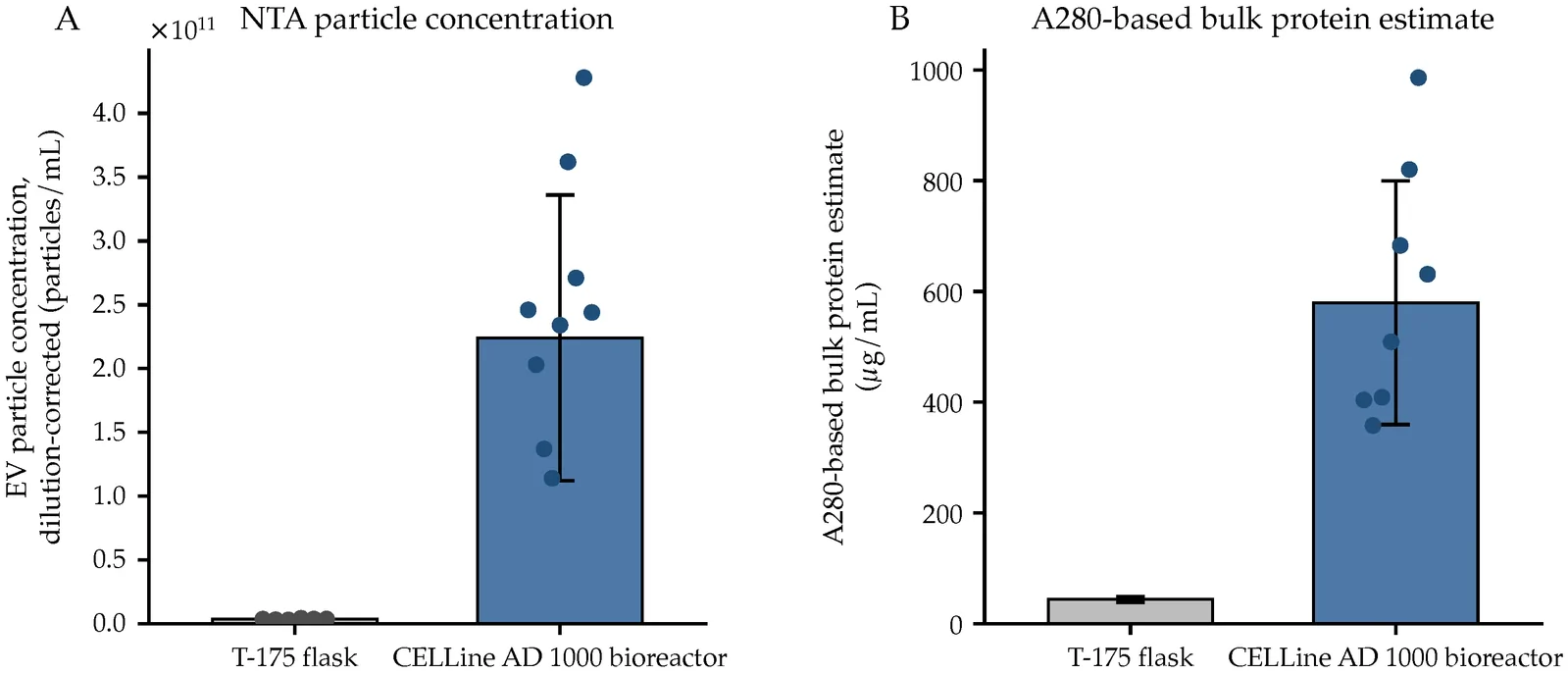
Comparison of EV recovery from OVCAR4 in a bioreactor and a flask comparator. (**A**) Dilution-corrected NTA particle concentration and (**B**) A280-based bulk protein estimate from OVCAR4 were compared between one CELLine AD 1000 bioreactor and one conventional T-175 flask. The flask comparator was maintained in the original pre-adaptation medium rather than matched serum-free CDM-HD conditions. Bars represent the mean ± SD across harvests, and individual dots represent measurements from each harvest. The data represent within-run variability across repeated harvests and were not subjected to inferential statistical testing.

To compare EV recovery between bioreactor and conventional flask culture, one OVCAR4 CELLine AD 1000 bioreactor was compared with one standard T-175 flask comparator (Figure 2). For each collection, 15 mL of conditioned medium was harvested from each system. The CELLine AD 1000 bioreactor samples showed nearly two orders of magnitude higher EV particle concentration and more than one order of magnitude higher A280-based bulk protein estimate than the T-175 flask comparator over the measured collections (Table 1). The bioreactor was harvested twice weekly, whereas the flask comparator was harvested once weekly; therefore, the values reported here reflect per-collection concentration differences rather than weekly cumulative EV production over time. Because culture format, medium formulation, and cell density were not independently controlled, the observed difference in EV concentration cannot be attributed specifically to CDM-HD, serum-free culture conditions, differences in cell density, or any other single factor.

### 3.3. Imaging Flow Cytometry Shows Differential CD9 and CD63 Positivity Across EVs

**Figure 3:**
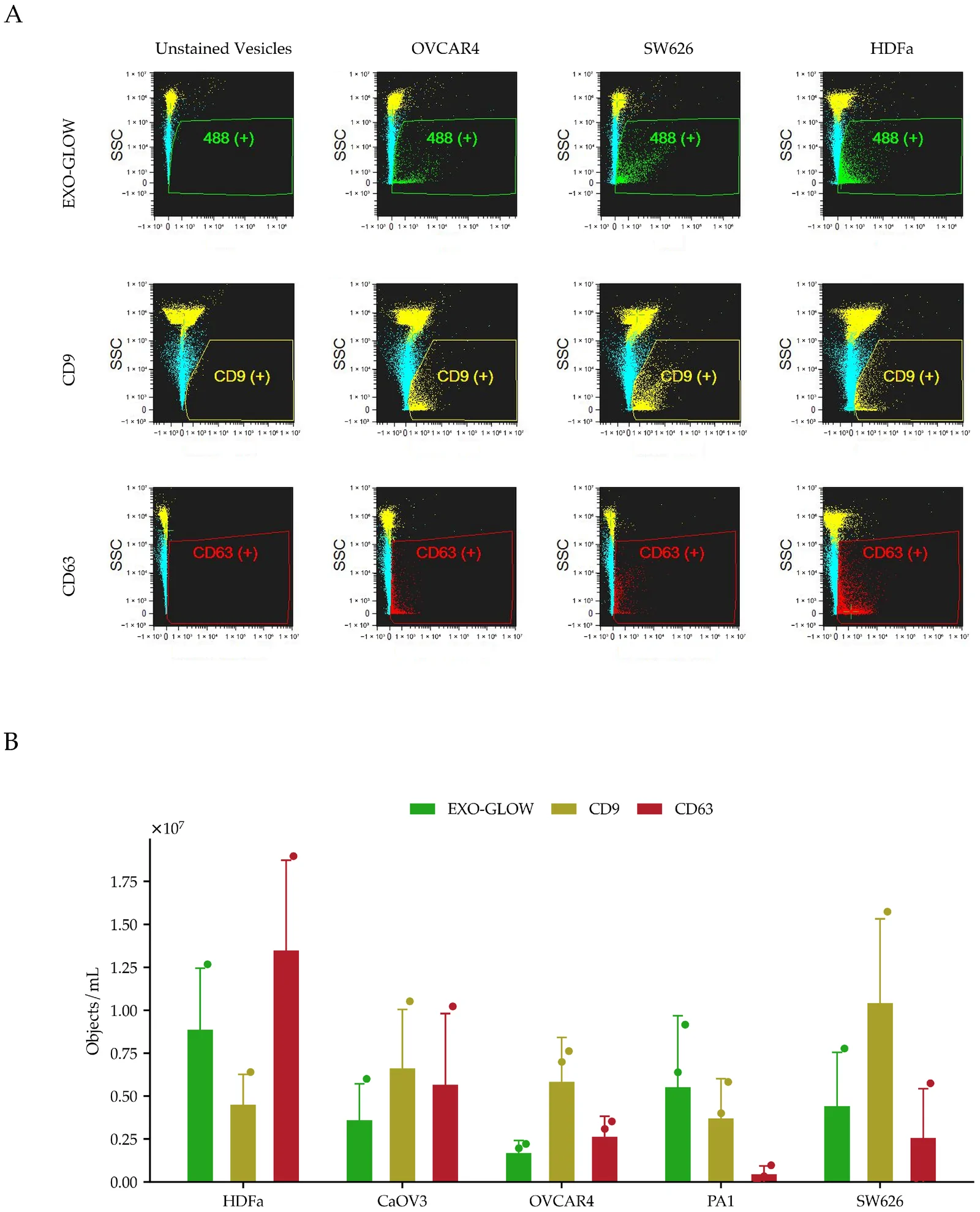
Imaging flow cytometry of CD9 and CD63 in EVs. (**A**) Representative ImageStream dot plots of side scatter (SSC) versus fluorescence intensity for, from left to right, an unstained vesicle control and OVCAR4, SW626, and HDFa EVs. Rows show the gating for EXO-GLOW-positive events (488/FITC channel, **top**), CD9-positive events (**middle**), and CD63-positive events (**bottom**). Each dot represents an individual detected event, and the green, yellow, and red gate outlines and labels indicate the EXO-GLOW-positive, CD9-positive, and CD63-positive regions, respectively. (**B**) Concentrations of EXO-GLOW-positive, CD9-positive, and CD63-positive events (objects/mL) across HDFa and the four OC-related cell lines (CaOV3, OVCAR4, PA1, and SW626). Green, yellow, and red bars represent EXO-GLOW-positive, CD9-positive, and CD63-positive events, respectively. Bars and error bars represent the mean ± SD, and the overlaid dots represent the three individual technical measurements of the same sample for each cell line. CD9-positive event concentrations exceeded CD63-positive event concentrations in the OC-related cell lines, whereas CD63-positive event concentrations were highest in HDFa.

EVs were analyzed for CD9 and CD63 expression by ImageStream imaging flow cytometry. Representative dot plots of side scatter versus fluorescence intensity are shown for an unstained vesicle control and for OVCAR4, SW626, and HDFa EVs (Figure 3A, left to right), with the unstained control used to set the gates for EXO-GLOW, CD9-positive, and CD63-positive events. Quantification across the five cell lines (Figure 3B) showed higher CD9-positive than CD63-positive event concentrations in the OC-related EVs (CaOV3, OVCAR4, PA1, SW626), whereas CD63-positive events were highest in HDFa. For each sample, event concentrations were calculated as the mean of three technical measurements.

### 3.4. Western Blot Detection of CD9 and CD63 in EV and Cell Lysates

**Figure 4:**
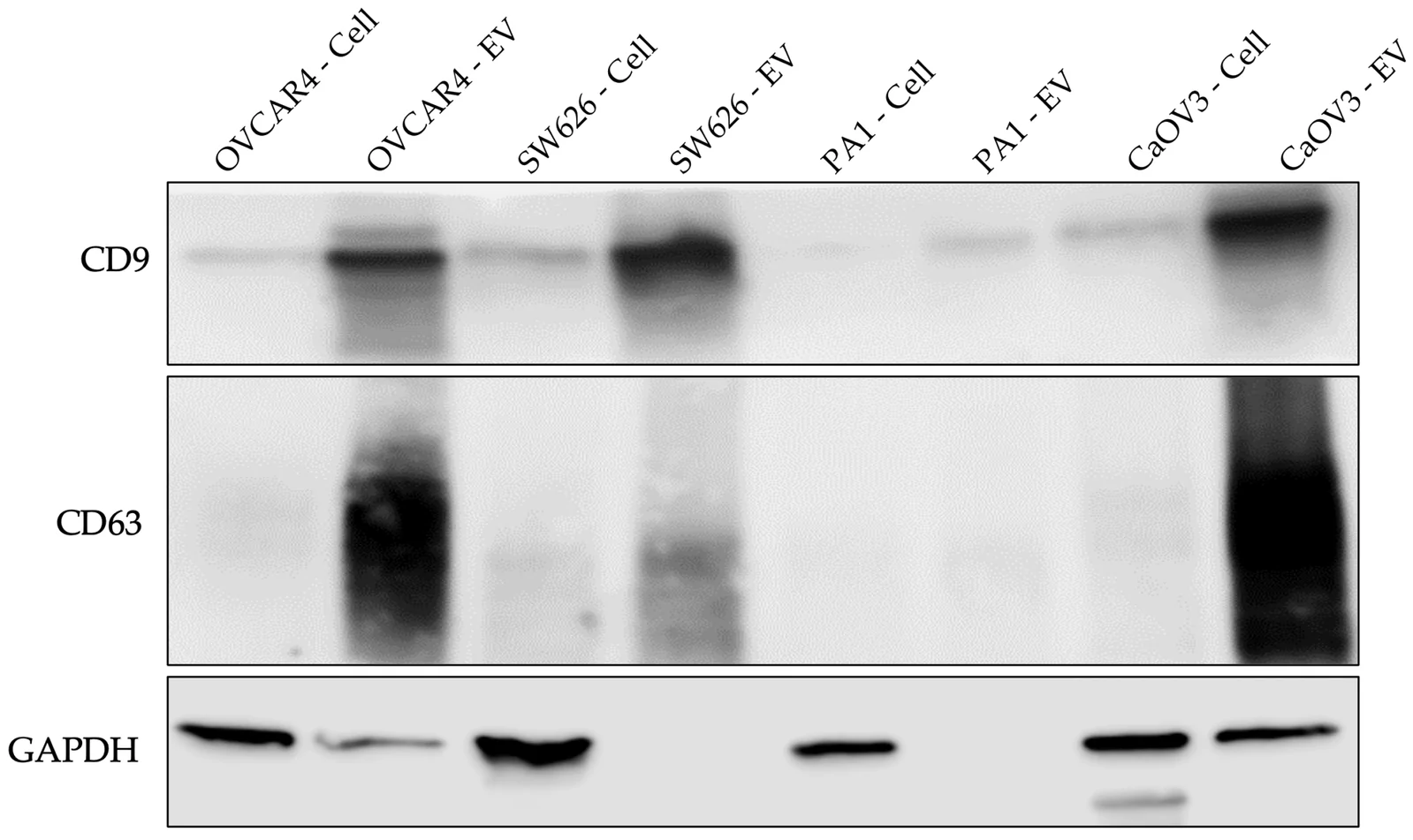
Western blot detection of CD9 and CD63 in EV and cell lysates. Representative blots show CD9 (**top**), CD63 (**middle**), and GAPDH (**bottom**) in paired cell lysate (-C) and EV lysate (-EV) samples from OVCAR4 (OV4), SW626, PA1, and CaOV3. For OVCAR4, SW626, and CaOV3, CD9 was detected as a band that was stronger in the EV lane than in the paired cell lysate, and CD63 was detected as a diffuse band in the same EV lanes. The PA1 lanes showed weak CD9 and little detectable CD63 signal. GAPDH was included as a loading reference and was detected mainly in the cell lysate lanes.

Paired EV and cell lysates from OVCAR4 (OV4), SW626, PA1, and CaOV3 were analyzed by Western blotting for CD9 and CD63 (Figure 4). For OVCAR4, SW626, and CaOV3, CD9 was detected as a band that was stronger in the EV lysate lane than in the paired cell lysate, with the most intense CD9 signal in the OVCAR4, SW626, and CaOV3 EV lanes. CD63 was detected as a broad, diffuse band in the same EV lysates, with the strongest signal in the OVCAR4 EV lane and weaker signals in the SW626 and CaOV3 EV lanes. The PA1 lanes showed weak CD9 and little detectable CD63 signal in both the cell and EV lysates. GAPDH, included as a loading reference, was detected mainly in the cell lysate lanes and was low in the EV lysate lanes; the CaOV3 cell lysate also showed an additional band below the main GAPDH band.

### 3.5. TEM Morphology and NTA-Based Particle Size Characterization

**Figure 5:**
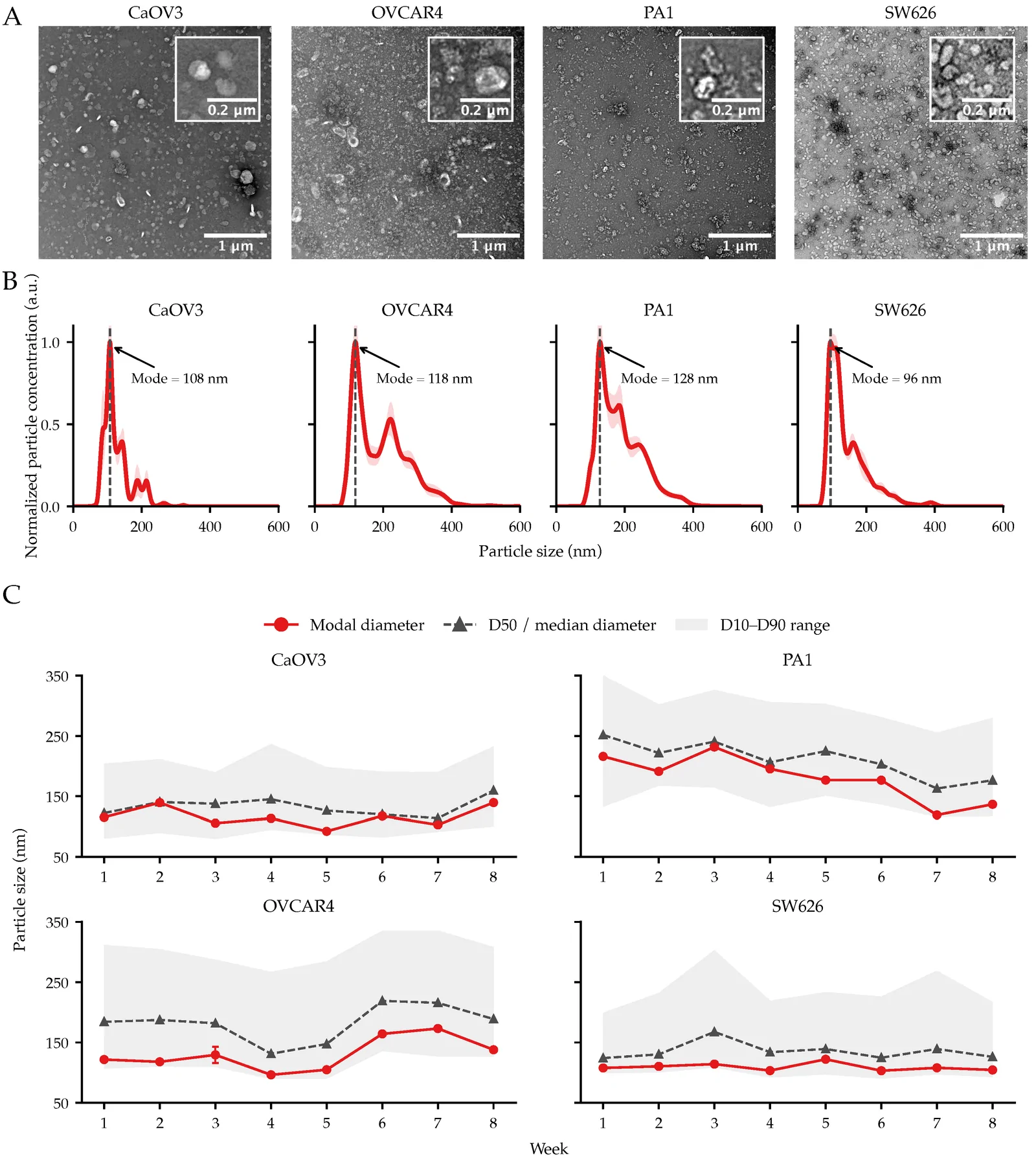
Morphology and NTA-based particle size characterization of ovarian cancer-related EV-enriched preparations. (**A**) Representative negative-stain TEM images of EV-enriched preparations from CaOV3, OVCAR4, PA1, and SW626. Scale bars indicate 1 µm in the main images and 200 nm in the insets. (**B**) Representative NTA size-distribution profiles for each cell line. The red curves represent the normalized particle-size distributions obtained by NTA, and the vertical dashed lines indicate the modal particle diameters. These profiles are shown as representative distributions and were not necessarily obtained from the same harvests as the TEM images. (**C**) NTA-derived modal diameter, D50 median diameter, and D10–D90 particle size range for one representative harvest per week across the eight-week bioreactor production period. The shaded region represents the D10–D90 percentile range and does not indicate measurement error.

Negative-stain TEM was used to examine the morphology of EV-enriched preparations from the four OC-related cell lines, CaOV3, OVCAR4, PA1, and SW626. Vesicle-like particles were observed in all four samples, with rounded morphology and heterogeneous particle sizes in the representative fields (Figure 5A). Higher-magnification insets showed individual nanoscale particles, including particles with a darker peripheral rim and lighter central region. Local particle clustering was also observed in some fields, particularly in CaOV3, PA1, and SW626.

NTA was then used to determine particle size distribution. The normalized size-distribution curves showed modal diameters of 108 nm for CaOV3, 118 nm for OVCAR4, 128 nm for PA1, and 96 nm for SW626 (Figure 5B). CaOV3 and SW626 showed dominant peaks below 150 nm, whereas OVCAR4 and PA1 showed broader distributions with additional signal extending toward larger particle sizes. For one representative harvest per week, the modal diameter, D50/median diameter, and D10–D90 range were plotted for each cell line (Figure 5C). CaOV3 and SW626 showed relatively compact size profiles across these weekly representative measurements, while OVCAR4 and PA1 showed broader D10–D90 ranges and greater variation in median particle size. Overall, EV particle modes across the four OC-related cell lines ranged from 96 to 128 nm.

### 3.6. SEM of Cell Coverage and Growth Morphology on Bioreactor Membranes

**Figure 6:**
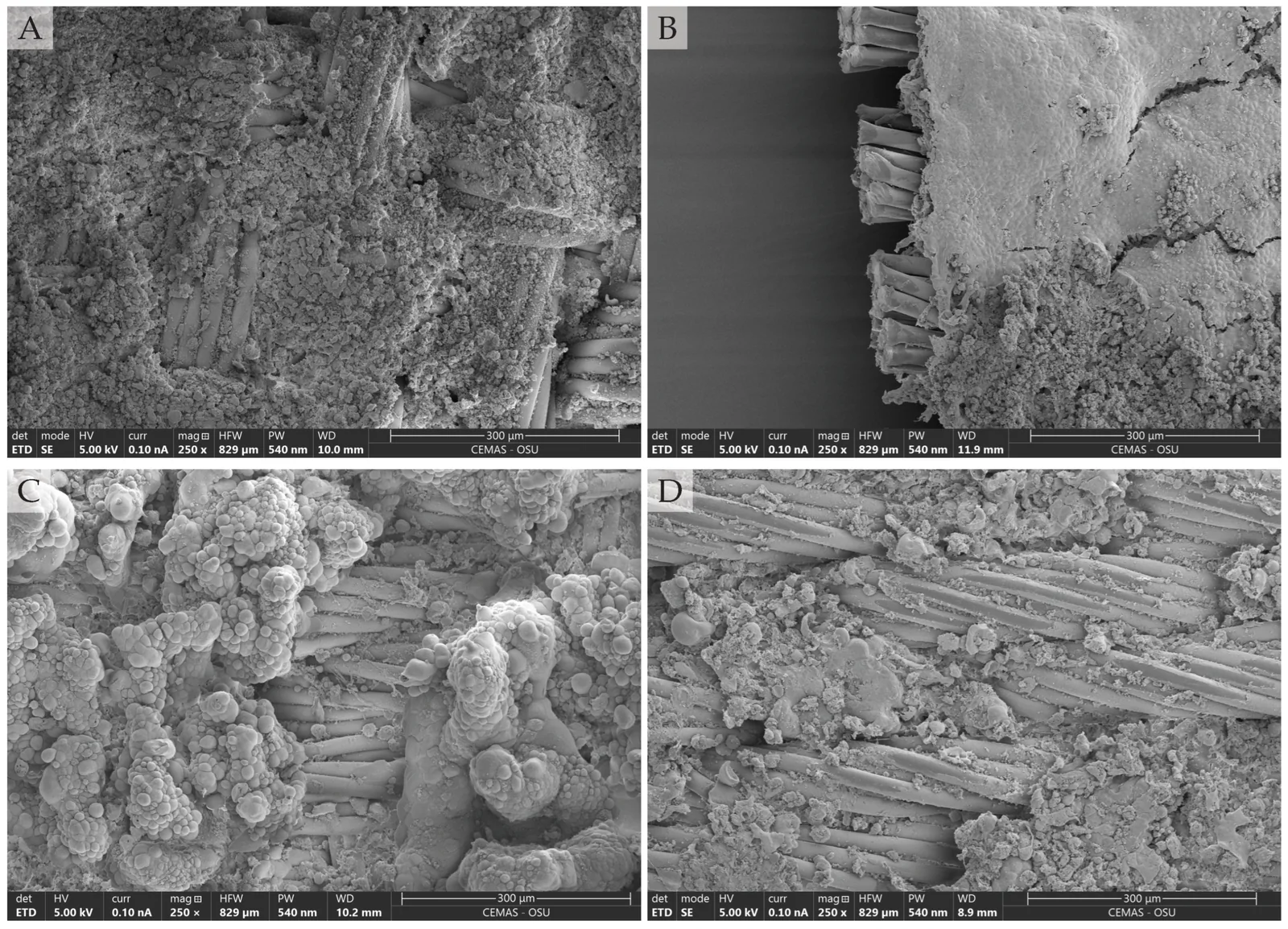
SEM of cells on CELLine AD 1000 bioreactor membranes after eight weeks of culture. Representative images of (**A**) CaOV3, (**B**) PA1, (**C**) OVCAR4, and (**D**) SW626 cells cultured on bioreactor membranes. CaOV3 showed broad coverage of the woven membrane surface, with abundant granular cell-associated material and partially exposed parallel support fibers. OVCAR4 displayed densely packed, rounded, mound-like three-dimensional clusters and aggregate-like structures overlying the membrane. PA1 showed a relatively continuous cell-covered surface near the membrane edge, with parallel support fibers visible along the edge. SW626 showed an elongated, fiber-associated growth pattern, with cell-associated material distributed along and between exposed parallel support fibers. Images were acquired at 250× magnification using ETD secondary-electron mode at 5.00 kV; scale bars represent 300 µm.

SEM imaging of CELLine AD 1000 membranes after eight weeks of culture showed cell growth and coverage on the membranes by all four OC-related cell lines, with line-specific surface morphologies in the representative fields examined (Figure 6). CaOV3 cells (Figure 6A) covered much of the woven membrane surface, with parallel support fibers still visible beneath and between areas of cellular material. OVCAR4 cells (Figure 6C) formed densely packed rounded clusters and aggregate-like structures, giving the membrane surface a nodular three-dimensional appearance. PA1 cells (Figure 6B), shown near the membrane edge, formed a relatively continuous layer over the membrane surface, while parallel support fibers were visible along the membrane edge. SW626 cells (Figure 6D) showed a fiber-associated growth pattern, with cell-associated material distributed over and between the aligned support fibers, which remained partly exposed. In these representative images, large uncovered membrane regions were not apparent, and each cell line showed a distinct endpoint appearance on the bioreactor membrane.

## 4. Discussion

Many benefits have been reported for the use of bioreactor systems in large-scale extracellular vesicle production, particularly in their ability to support high-density cell culture, reduce manual handling, and generate concentrated conditioned media for downstream EV isolation [27,31,32,33,34,35,36,39]. These features are especially relevant for ovarian cancer EV research, where sufficient cell-line-derived material is needed for assay development, molecular profiling, and mechanistic studies. In this study, we demonstrate that a serum-free CELLine AD 1000 workflow can be applied to multiple ovarian cancer-related cell lines and controls and can support repeated EV particle collection over an extended culture period.

Practically, the CELLine AD 1000 format offers several advantages compared with conventional flask-based culture. The separation of the cell compartment from the nutrient reservoir allows cells to be maintained at high density while EVs remain concentrated in a relatively small harvest volume. This reduces the culture footprint and avoids the need to process large volumes of dilute conditioned medium before EV isolation. In the OVCAR4 comparison, the bioreactor-derived samples had a higher per-collection particle concentration than the conventional flask samples. This difference may reflect the higher cell density supported by the bioreactor and the accumulation of secreted particles within its relatively small cell compartment. However, because serum-free DMEM/F12 supplemented with CDM-HD was used in the bioreactor whereas RPMI-1640 supplemented with FBS was used in the flask, medium composition was confounded with culture format. Therefore, the present comparison cannot establish a medium-specific advantage, and a matched comparison of both media in both culture formats would be required to distinguish these effects. Rather than simply increasing culture volume, the system creates a compact production environment in which repeated harvests can be obtained with lower handling requirements. Importantly, the ability to obtain repeated harvests from a single culture system may improve workflow consistency and reduce variability associated with repeated flask expansion procedures. From a biomanufacturing perspective, this feature is particularly attractive because it enables sustained EV production while reducing labor demands, culture footprint, and upstream processing complexity. These findings are consistent with previous studies demonstrating similar improvements in EV yield using bioreactor-based culture systems for mesenchymal stromal cells (MSCs), HEK293 cells, and cancer cell lines, supporting the broader applicability of bioreactor-based EV production strategies [32,33,34,35,36,38,39]. Beyond comparisons with conventional flask culture, bioreactor selection involves a balance between EV output and operational simplicity. In a recent direct comparison, a hollow-fiber bioreactor produced a higher mean EV particle output per unit volume of harvested medium than the CELLine AD 1000 under the conditions tested [39]. However, hollow-fiber systems require continuous medium circulation and dedicated perfusion hardware, whereas the CELLine AD 1000 can be seeded, maintained, and harvested using procedures similar to standard flask culture in conventional biosafety cabinets and incubators [27,31,39]. Thus, the CELLine AD 1000 combines the accessibility of routine flask-based culture with concentrated, repeated EV collection and reduced handling compared with scaling through multiple conventional flasks.

The serum-free CDM-HD adaptation is also central to the utility of this workflow. Serum-containing media can introduce exogenous vesicles and soluble proteins that complicate interpretation of EV yield, marker abundance, and downstream molecular analyses [29,30]. By transitioning the cultures to a defined serum-replacement condition, the workflow provides a cleaner production setting for cell-line-derived EV studies [38,43]. When combined with differential ultracentrifugation and size-exclusion chromatography, this approach provides a practical and reproducible isolation sequence for obtaining EV-enriched material suitable for orthogonal characterization [41,44]. However, because chemically defined and serum-containing media were not compared longitudinally under matched conditions, the present study cannot determine whether medium type has a time-dependent effect on EV properties.

The production patterns observed across the ovarian cancer-related cell lines further suggest that EV output is strongly influenced by parental cell identity. The OC-related cultures produced higher EV particle and bulk protein output than HDFa, while individual cancer-related cell lines displayed distinct longitudinal production profiles. These differences are consistent with the broader concept that EV release is not solely a function of culture format, but also reflects cell-intrinsic biology, growth behavior, and adaptation to the bioreactor environment. For ovarian cancer-related models, this is an important consideration because the disease itself is heterogeneous, and EV preparations derived from different cellular backgrounds may retain distinct physical and molecular properties.

SEM imaging provides useful context for interpreting these production patterns. The OC-related cell lines formed distinct growth architectures on the CELLine membrane, including clustered, layered, and fiber-associated morphologies. Similar observations in other CELLine AD 1000 studies have suggested that bioreactor growth can generate dense, three-dimensional, tissue-like cellular arrangements that differ from conventional two-dimensional monolayer culture [27,31]. In the present study, the distinct membrane-associated growth patterns of CaOV3, OVCAR4, PA1, and SW626 may help explain why EV output and particle characteristics varied among cell lines grown under the same production workflow. The bioreactor therefore appears to function not only as a higher-output culture platform, but also as a biologically relevant microenvironment capable of preserving cell-line-specific growth architecture and EV production characteristics.

The EV characterization data support the recovery of vesicle-like particles from this workflow. TEM demonstrated EV-like morphology, while NTA showed nanoscale particle distributions across the ovarian cancer-related preparations. The broader size distributions observed in OVCAR4 and PA1 are consistent with the known heterogeneity of EV-enriched isolates and with the possibility that different cell lines release different proportions of vesicle subpopulations. The observed variability in particle size distribution and tetraspanin expression highlights the continuing challenge of EV and cancer cell heterogeneity. Bioreactor systems may increase production efficiency, but they do not eliminate the need for rigorous characterization to distinguish between EV subpopulations and potential non-vesicular contaminants. These findings support the use of complementary methods when evaluating bioreactor-derived EV material, as morphology, size distribution, and marker analysis from each capture different aspects of EV preparation quality [26,45].

Tetraspanin profiling further showed that the generated EVs retain measurable EV-associated marker features while also reflecting cell-source-dependent variation. Imaging flow cytometry detected differential CD9 and CD63 positivity across EVs, and Western blotting confirmed CD9 and CD63 signals in OC-related EV lysates. The stronger CD9 pattern in OC-related EVs and the prominent CD63 signal in HDFa-derived particles suggest that tetraspanin abundance is not uniform across cell sources. Rather than defining a single ovarian cancer EV signature, these results highlight the importance of characterizing each EV-producing cell line individually. Such differences may reflect variation in EV subpopulation composition, vesicle biogenesis routes, or parental cell phenotype [46,47]. Tetraspanins such as CD63 have also been reported as candidate biomarkers in ovarian cancer patients [48], underscoring the value of characterizing tetraspanin profiles in EV material derived from individual cell lines.

Together, the particle-yield, morphology, tetraspanin, and membrane-growth findings indicate that the CELLine AD 1000 system is a useful platform for producing characterized ovarian cancer-related EV material. The workflow generated sufficient EV-enriched preparations for multiple analytical methods while preserving differences among cell lines. This is valuable for studies that require parallel comparison of EVs from distinct ovarian cancer-related models, as well as for downstream applications in biomarker-oriented assay development, cargo profiling, and functional EV research. Accordingly, the present study addresses an upstream bioengineering need by providing a production and characterization workflow for ovarian cancer-related EV studies, rather than directly evaluating diagnostic or therapeutic performance.

This study has some limitations. It was conducted in vitro using a single bioreactor per cell line; the absence of biological replication limits statistical comparison of EV production metrics, so the comparisons reported here are descriptive. Protein content was estimated as a bulk measure by A280, and molecular cargo profiling was not performed, preventing assessment of whether bioreactor culture influences EV protein, RNA, or other cargo composition relative to conventional culture conditions. Confirmation of these findings will require independent production runs, additional orthogonal characterization methods, and functional validation studies.

Although the current study focused on ovarian cancer models, scalable EV production remains a key challenge for biomarker development and future therapeutic applications across other cancer types and diseases. Bioreactor-based workflows may help address limitations associated with conventional culture systems by improving production efficiency while reducing labor and culture footprint. Future studies should evaluate biological reproducibility across independent production runs, investigate the molecular cargo of bioreactor-derived EVs, and determine whether EV functional activity is maintained during long-term bioreactor culture. Extending these studies across additional ovarian cancer subtypes may also provide insight into how tumor heterogeneity influences EV production and composition.

Collectively, these findings support the CELLine AD 1000 bioreactor as a practical serum-free production platform for ovarian cancer-related EV studies. The system addresses a major practical barrier in EV research by increasing available EV material while maintaining compatibility with standard characterization workflows. As interest in tumor-derived EVs continues to grow, this workflow provides a useful foundation for generating EVs from ovarian cancer-related cell lines in a format suitable for downstream analytical and translational applications.

## 5. Conclusions

This study shows that the CELLine AD1000 bioreactor can support repeated serum-free production of EVs from OC-related cell lines over an eight-week culture period. In descriptive comparisons, the bioreactor produced nearly two orders of magnitude higher EV concentration output than a conventional T-175 flask comparator, although the flask was not maintained under matched serum-free conditions. The resulting EVs showed vesicle-like morphology and detectable CD9/CD63 tetraspanin patterns by complementary characterization methods. Because one bioreactor was used per cell line, these findings should be interpreted as preliminary and descriptive. Future studies using independent bioreactor replicates, matched flask controls, expanded EV marker panels, cargo profiling, functional assays, and patient-derived samples will be needed to further evaluate the utility of this workflow for ovarian cancer EV studies.

## Figures and Tables

**Table 1:** EV particle concentration and A280-based bulk protein estimate for OVCAR4 produced in one CELLine AD 1000 bioreactor and one T-175 flask comparator. Values are mean ± SD across harvests.

OVCAR4	Particle Concentration (Particles/mL, Mean ± SD)	Bulk Protein Estimate (µg/mL, Mean ± SD)
One T-175 flask	$3.59\times 10^{9}\pm 4.84\times 10^{8}$	43.83±5.38
CELLine AD 1000 bioreactor	$2.49\times 10^{11}\pm 9.90\times 10^{10}$	600.00±222.64

## Data Availability

The data presented in this study are available on request from the corresponding author.

## Abbreviations

The following abbreviations are used in this manuscript:

EV	extracellular vesicle
sEV	small extracellular vesicle
OC	ovarian cancer
HDFa	human dermal fibroblasts (adult)
FBS	fetal bovine serum
CDM-HD	chemically defined medium—high density (serum replacement)
SEC	size-exclusion chromatography
NTA	nanoparticle tracking analysis
TEM	transmission electron microscopy
SEM	scanning electron microscopy
PBS	phosphate-buffered saline
MISEV2023	Minimal Information for Studies of Extracellular Vesicles 2023
ISEV	International Society for Extracellular Vesicles
HGSC	high-grade serous carcinoma
CEMAS	Center for Electron Microscopy and Analysis
